# Methodology for a fully automated pipeline of AI-based body composition tools for abdominal CT

**DOI:** 10.1007/s00261-025-04951-7

**Published:** 2025-04-28

**Authors:** John W. Garrett, Perry J. Pickhardt, Ronald M. Summers

**Affiliations:** 1 Department of Radiology, University of Wisconsin School of Medicine & Public Health, Madison, WI, USA; 2 Department of Medical Physics, University of Wisconsin School of Medicine & Public Health, Madison, WI, USA; 3 Department of Biostatistics and Medical Informatics, University of Wisconsin School of Medicine & Public Health, Madison, WI, USA; 4 Imaging Biomarkers and Computer-Aided Diagnosis Laboratory, Department of Radiology and Imaging Sciences, National Institutes of Health Clinical Center, Bethesda, MD, USA

**Keywords:** Abdomen, CT, Deep learning, Machine learning, Opportunistic Screening

## Abstract

Accurate, reproducible body composition analysis from abdominal computed tomography (CT) images is critical for both clinical research and patient care. We present a fully automated, artificial intelligence (AI)-based pipeline that streamlines the entire process—from data normalization and anatomical landmarking to automated tissue segmentation and quantitative biomarker extraction. Our methodology ensures standardized inputs and robust segmentation models to compute volumetric, density, and cross-sectional area metrics for a range of organs and tissues. Additionally, we capture selected DICOM header fields to enable downstream analysis of scan parameters and facilitate correction for acquisition-related variability. By emphasizing portability and compatibility across different scanner types, image protocols, and computational environments, we ensure broad applicability of our framework. This toolkit is the basis for the Opportunistic Screening Consortium in Abdominal Radiology (OSCAR) and has been shown to be robust and versatile, critical for large multi-center studies.

**Graphical abstract:**

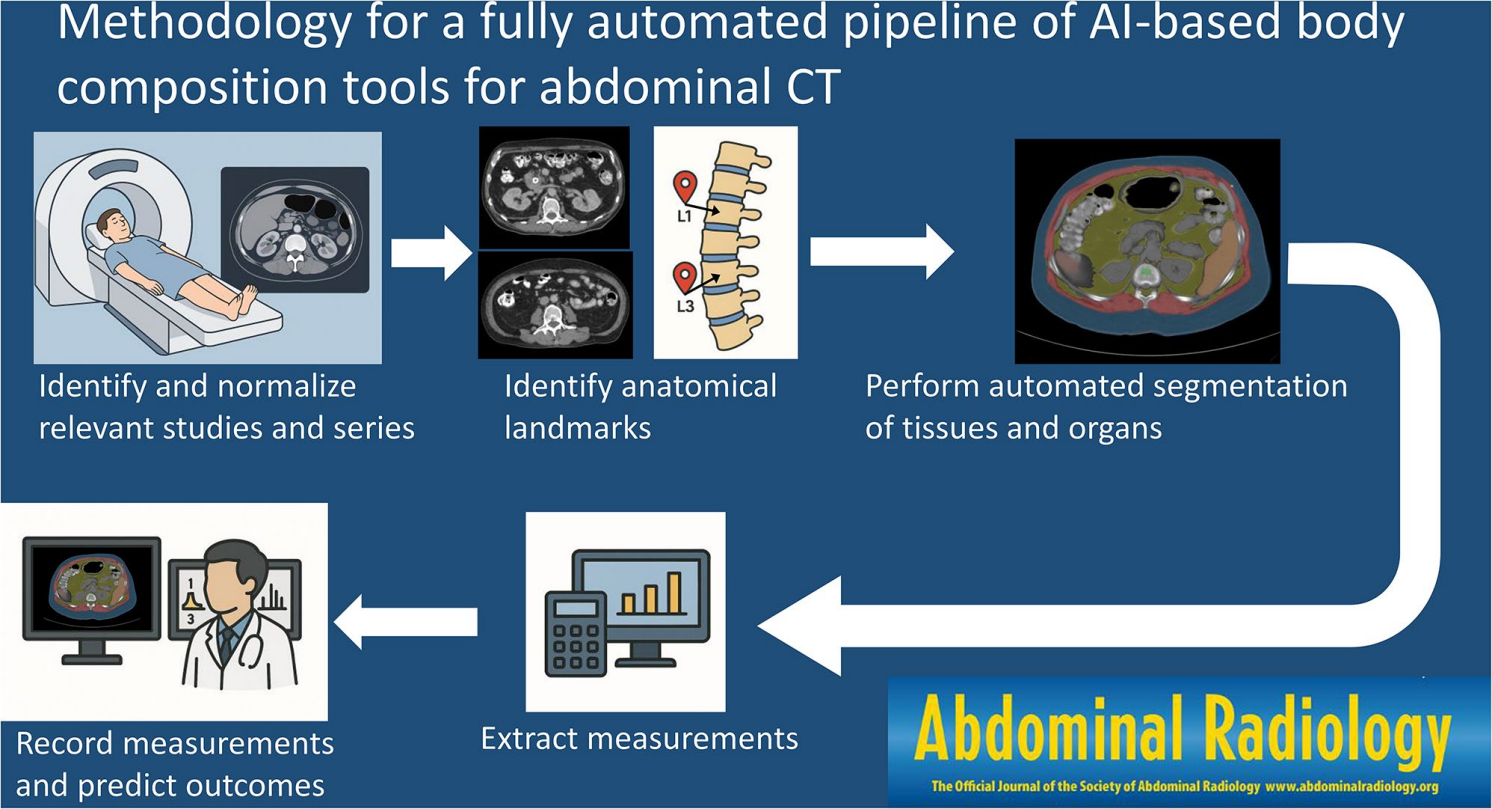

## Introduction

The World Health Organization defines screening as a process aimed at identifying individuals within a seemingly healthy population who are at an elevated risk for certain health conditions. This early identification allows for timely intervention or treatment, potentially reducing the incidence and mortality associated with these conditions. In medical practice, opportunistic screening refers to preventive measures taken during unplanned or incidental encounters. In radiology, this concept has evolved to include the systematic use of imaging data obtained for reasons unrelated to the primary clinical indication.

A notable example of this is the quantitative analysis of body composition data from CT scans of the abdomen or thorax. By measuring various tissues and organs such as muscle, fat, bone, liver, spleen, kidneys, and arterial calcification, these scans can provide valuable prognostic information for risk stratification or early disease detection. While opportunistic screening can be applied to other imaging modalities like conventional radiography, ultrasound, and MRI, the focus has predominantly been on body CT, especially with the advent of AI-assisted methods. The growing interest in population-based opportunistic CT screening is driven by several factors: the objective nature of CT measurements, the comprehensive cross-sectional imaging of the torso, reproducibility across different CT vendors [1], the high volume of CT scans performed annually [2], the development of explainable and interpretable AI algorithms along with a rapid rise in available computational resources, especially graphical processing units (GPUs) [3, 4], and the increasing emphasis on enhancing the value of routine radiology practice [5].

CT images, and those from other imaging modalities, contain a wealth of data beyond their primary clinical purpose, which can have both positive and negative implications. Historically, the focus has been on the potential negative consequences of incidental findings, or “incidentalomas” [6]. However, the potential benefits of these incidental findings are significant. When fully utilized, the incidental data from CT scans can offer a net benefit to patients. Major public health issues such as cardiovascular disease, osteoporosis, metabolic syndrome, sarcopenia, and cancer account for the majority of deaths in our aging population. Detecting these conditions early in asymptomatic individuals at higher risk could lead to better clinical outcomes through timely lifestyle changes or medical interventions [7–10].

CT scans of the chest or abdomen can provide a comprehensive evaluation of tissues and organs beyond the initial clinical indication for imaging. Examples of quantitative assessments include bone mineral density for osteoporosis, visceral fat for metabolic syndrome, muscle mass for sarcopenia, liver fat for steatosis, and arterial calcification for atherosclerosis. Additional assessments can detect conditions like hepatic fibrosis, cirrhosis, diabetes, and urolithiasis. Studies on CT colonography have shown that detecting unsuspected conditions such as osteoporosis, abdominal aortic aneurysms, and extracolonic cancers can enhance both the clinical efficacy and cost-effectiveness of the examination [11].

Emerging evidence suggests that AI-assisted body composition analysis in opportunistic CT screening for osteoporosis and cardiometabolic diseases can improve clinical outcomes and be cost-effective, potentially even cost-saving. The development of fully automated, explainable AI tools for body composition analysis marks a significant advancement in opportunistic screening. These tools have demonstrated the potential to predict adverse clinical outcomes, including major cardiovascular events, osteoporotic fractures, and mortality. In this work, we outline how to develop automated segmentation tools for body composition measurement, assemble pipelines that are both portable and robust, and identify strategies for clinical integration. To illustrate these concepts, we highlight the use of a well-validated pipeline currently employed by the Opportunistic Screening Consortium in Abdominal Radiology (OSCAR), a global group of academic and private radiology practices conducting research in opportunistic CT screening and body composition analysis [8].

### Body composition analysis pipeline

**Fig. 1:**
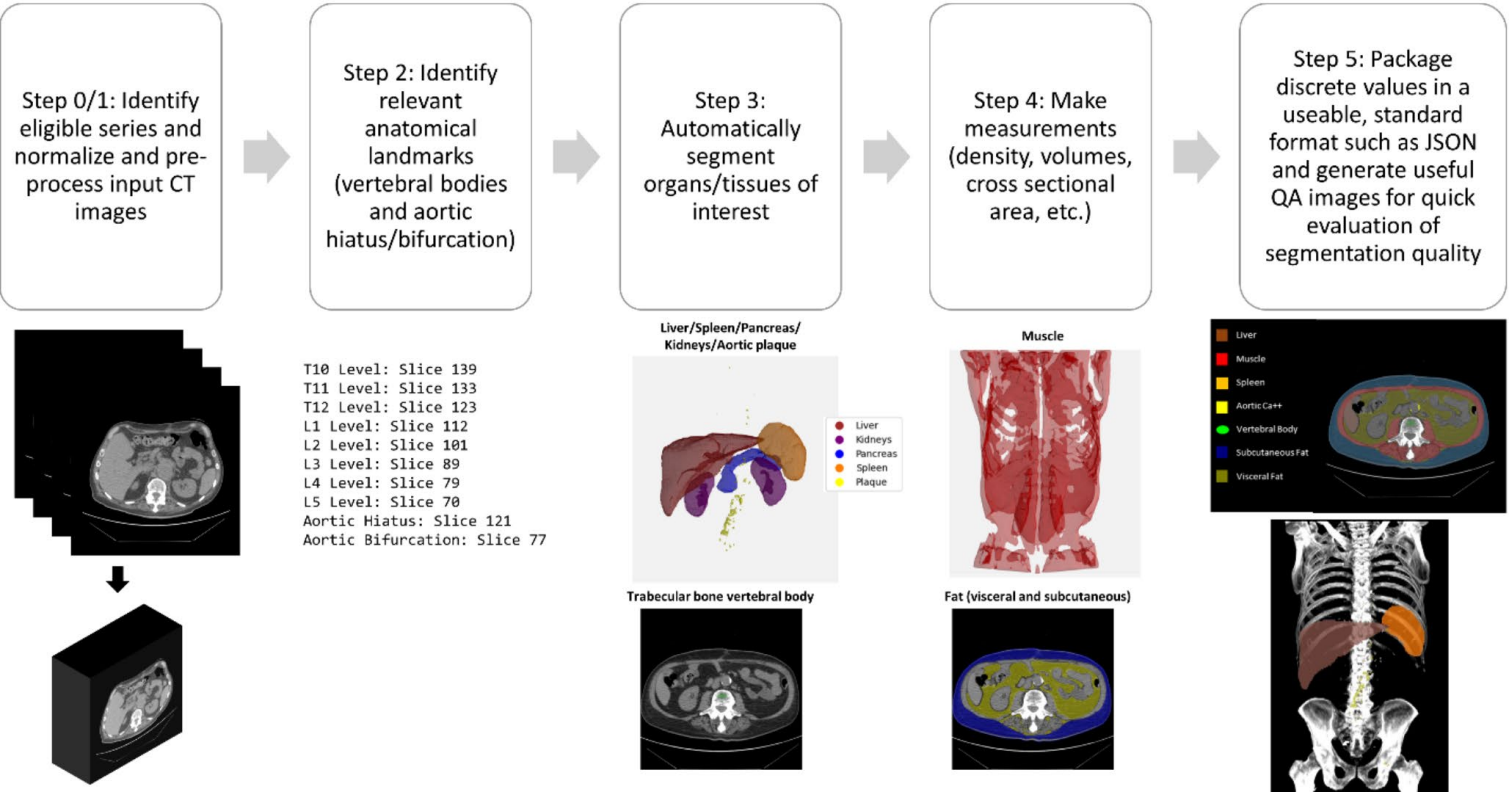
A diagram of the OSCAR toolkit showing the 5 major steps of the processing pipeline

Automated body composition analysis has been performed by many different groups and various toolkits exist for academic purposes and more recently clinical use [12]. Whether academic or commercial, these pipelines need to be automated and reproducible to be useful. Each of these pipelines assume that a correct and relevant series has been identified already by an orchestration/routing engine or in some cases filter irrelevant ones out on arrival. Once the correct series is identified, most of these pipelines will follow five steps listed below and shown in Fig. 1:

1. Data are normalized and preprocessed to provide consistent inputs to the subsequent components of the pipelines.
2. Some type of localization or anatomical check is performed to ensure relevant anatomy is present in the series. This is often a step that may trigger an exit from the pipeline if relevant anatomy isn’t present rather than passing irrelevant images to subsequent steps.
3. Using a variety of techniques and tools ranging from rules based or thresholding methods to state-of-the-art deep learning methods, organs and tissues of interest are automatically segmented.
4. Those segmentations are used to generate measurements from the study such as volumes, cross-sectional areas, or attenuation/CT Number measures.
5. These results (discrete measurements and image segmentations) are packaged up in a useful, portable, standards-based output that can be shared with downstream tools such as the electronic medical record (EMR) or radiology reporting software.

### Data normalization and anatomical landmarking

Data preprocessing and normalization is a critical step that helps alleviate some of the variability seen due to different CT protocols, vendor specific Digital Imaging and Communications in Medicine (DICOM) file attributes, or patient orientation. The first step in the OSCAR toolkit is to produce a single portable NIFTI file from the input DICOM images using the dcm2niix application (Version 1.0.20181125-1build1) and applying intensity scaling to ensure CT numbers are consistent across different scans. The intensity scaling is achieved subtracting the minimum value of the generated NIFTI such that background and air values are 0 avoiding negative values. The DICOM to NIFTI conversion may need to accommodate a wide range of protocols, including series where multiple contrast phases are included as a single series [13] or irregular slice sampling. The issue of multiple acquisitions per series is accommodated by taking the set of DICOM images (a set is defined as all images for a given acquisition number; DICOM tag 0020,0012) with the greatest axial coverage. The acquisition number is only unique within a given series so this selection must be performed for a single series. If multiple acquisition numbers span the same axial distance, then the lowest acquisition number is used. The issue of irregular slice sampling is handled with a simple linear interpolation. The input NIFTI volume is then automatically reoriented if needed to match a standard head-first-supine patient position. The toolkit then resamples the input CT images to a standard voxel size of 3 mm thick with 3 mm spacing. This 3 × 3 spacing retains sufficient detail and limits partial volume artifacts without providing overly noisy images. This sampling also yields a manageably sized volume (in terms of disk space and RAM usage) that may be processed by a wide range of computational resources; this has been shown to be a good compromise that yields similar results for input images that are natively thin (< 1 mm slices) or thicker [14].

**Fig. 2:**
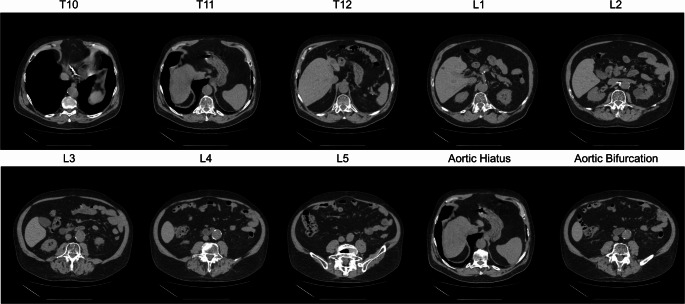
A representative case of a 74 year-old male patient demonstrating the levels identified in the processing pipeline for different anatomical landmarks

Once the volume has been normalized and standardized, typically an anatomical landmarking is performed. There are many ways this can be performed, in the OSCAR toolkit this is achieved using a body part regression tool [15]. This tool was trained in an unsupervised manner to return a “score” for each slice in an image that describes the anatomical location. A total of 800 unlabeled CT volumes were collected from 420 subjects. These volumes ranged from 30 to 700 slices and predominantly consisted of chest-abdomen-pelvis scans. No anatomical labels or scan range annotations were provided during training. The model was trained in a fully unsupervised manner, with anatomical region boundaries inferred solely from the image data. Manual slice-level labels in the chest, abdomen, and pelvis were applied only to the testing set for the purpose of quantitative evaluation. By presenting the model with many axial CT images and then calibrating the output scores with known relevant landmarks, the tool can reliably provide a slice position for landmarks of interest. In the OSCAR toolkit, the relevant landmarks are the 10th -12th thoracic vertebral bodies and 1st -5th lumbar vertebral bodies (defined at the center of the vertebral body; see Fig. 2 for an example case) as well as the aortic hiatus and bifurcation. These levels are used to define a standard measurement position for fat and muscle values [16] as well as to define the abdominal compartment of the aorta for calcified plaque measurement.

### Automated segmentation tools

The core components of any body composition toolkit are automated tissue segmentation tools that have been trained to identify and segment tissues and organs of interest. These segmentations will be used to automatically measure things like volume, cross sectional area, CT numbers, etc. Some of the desired measurements will be volumetric (e.g. abdominal aortic Agatston score or liver volume) while others are more commonly performed in a single slice (e.g., trabecular bone density at L1, visceral fat cross sectional area at L3, etc.). There may be minor inconsistencies in 2D models applied slice by slice so for segmentations that will be used for 3D measurements a 3D segmentation model should be used to ensure a consistent and useful segmentation.

Although 3D models can be applied to 2D tasks, it may not make sense to train 3D models for every segmentation task. 3D models may require up to 20 times the system RAM to be trained which may be unachievable or excessively expensive [17]. For certain tasks they may also not perform as well since assembling a large enough cohort of unique 3D samples for training is more difficult than for 2D training (there are many image slices per volume) [18].

In addition to choosing between 2D and 3D models, the specific model architectures may be useful or well suited to specific tasks. Some models are trained to do many things simultaneously, for example segment many different tissue classes at once. A well-known example of this is TotalSegmentator [19] which simultaneously segments more than 100 anatomical structures in CT exams using an nnUnet architecture [20]. These “segment everything” models are handy for things like organs or bony structures which have standard definitions, however if a specific sub compartment of a tissue (trabecular bone in a vertebral body or calcified plaque in a vessel for example) is needed or if tissue needs to be subclassified (visceral versus subcutaneous adipose tissue), then tailored models are needed for those specific tasks. For each of these tailored models, a dedicated training cohort will be needed with annotated images highlighting the desired tissue segmentations. In addition, a model architecture that is well suited to the specific task should be selected. Popular modern model architectures include variants of the U-Net [21] and vision transformer models (ViTs) [22].

To develop robust machine learning models in medical imaging, especially in CT segmentation tasks, it is essential to source high-quality labeled data. Curating such datasets often involves multiple approaches, including the use of public datasets, establishing data-sharing agreements, and implementing effective data labeling strategies. Public datasets like The Lung Image Database Consortium (LIDC) and Image Database Resource Initiative (IDRI), which contains annotated CT images for lung cancer screening, have been instrumental in supporting segmentation research [23]. Other notable sources include The Cancer Imaging Archive (TCIA) [24], which hosts a variety of labeled medical imaging datasets, and the American Association of Physicists in Medicine’s (AAPM) Radiotherapy MRI Auto-Contouring (RT-MAC) Challenge, which provides CT data for radiation therapy planning [25]. Beyond public datasets, data-sharing agreements between institutions, often governed by regulatory frameworks, enable the aggregation of diverse datasets while adhering to patient privacy requirements [26]. However, these agreements can be complex to navigate due to varying institutional policies and privacy regulations [27]. The high variability in CT image quality and anatomy across institutions further complicates efforts to achieve consistent labeling standards, a critical factor for training accurate segmentation models [28].

Data augmentation techniques play a vital role in enhancing the generalizability of labeled CT datasets, especially in medical imaging applications where labeled data can be limited. Techniques such as random rotation, scaling, cropping, and intensity variations simulate variations commonly encountered in clinical settings, thereby improving the model’s ability to generalize to new data [29]. More advanced approaches include synthetic data generation using generative adversarial networks (GANs), which can create realistic CT images that expand the training set and improve performance on segmentation tasks [30, 31]. These augmentation methods not only address data scarcity but also mitigate the challenges posed by class imbalance, which is common in medical imaging data. Moreover, techniques like semi-supervised learning, where a smaller set of labeled data is combined with a larger set of unlabeled data, have shown promise in CT segmentation by maximizing the utility of available data [32]. Through these approaches, researchers can create more robust models that better handle the complexities of real-world CT images.

The OSCAR pipeline includes automated segmentation of several abdominal organs including the liver, spleen, pancreas, and kidneys, as well as abdominal adipose tissue (semantically segmented into visceral and subcutaneous adipose tissue), muscle (including intramuscular adipose tissue), aortic calcification, and trabecular bone in the vertebral bodies. The model architectures, characteristics, and training cohorts are summarized in Tables 1, 2 and 3, and Table 4. Although this pipeline is in use at multiple academic sites, it is not published under an open license for public use (with the exception of the TotalSegmentator [19] component).

**Table 1:** Model architectures, approaches (slicewise vs. 3D volumetric), and weight size for each segmentation model

Target Tissue/Organ	Model Architecture	Slicewise or 3D measurement	Size of model weights (MB)
Vertebral trabecular bone [33]	TernausNet ((U-Net with VGG11 backbone)) [34]	Slicewise	87.5
Abdominal fat	TernausNet (UNet11 (U-Net with VGG11 backbone), modified final layer to output 3 classes [34]	Slicewise	87.5
Muscle	3D Swin Transformer Generator [35]	Slicewise	246.4
Aortic calcium	Swin UNETR [36]	3D	244.5
Liver, Spleen, Kidney, Pancreas	Total Segmentator 1.5.7 (nnUnet) [19]	3D	245.9

**Table 2:** Unique number of exams and patient demographics used to train each segmentation model

Target Tissue/Organ	Unique studies used for training and validation (Training/Validation Split %)	Patient/Scan Demographics			
		Male/Female (%)	Average Age +/- Std. Dev (Years)	Number of scanner manufacturers	IV Contrast Present/Absent (%)
Vertebral trabecular bone [33]	14,290 (80/20)	50/50	61.8 +/- 14.0	5	82/18
Abdominal fat	9,077 (80/20)	39/61	57.5 +/- 12.2	5	14/86
Muscle	21,084 (80/20)	63/37	57.3 +/- 12.5	6	29/71
Aortic calcium	7,879 (80/20)	61/39	68.4 +/- 10.5	5	39/61
Liver, Spleen, Kidney, Pancreas	1,204 (90/5/5(Test))	See paper for details			

**Table 3:** Data augmentation techniques used for model training

Target Tissue/Organ	Data Augmentation
Vertebral trabecular bone	Resize longest image side to 512 pixels Pad images to ensure 512 × 512 resolution Random shift (± 10%), scale (± 10%), rotation (± 185 degrees) with 50% probability Normalize pixel intensity with mean = 0.5, std = 0.25
Abdominal fat	Resize longest image side to 512 pixels Pad images to ensure 512 × 512 resolution Random shift (± 10%), scale (± 10%), rotation (± 185 degrees) with 50% probability Constant border padding (border_mode = cv2.BORDER_CONSTANT) with mask fill value (1,0,0) to preserve multi-class labels after augmentation Normalize pixel intensity (mean = 0.5, std = 0.25)
Muscle	Load images and labels; ensure channels first Intensity scaling normalized to [0,1] using window [-200, 200] Foreground cropping based on intensity threshold Reorientation to RAS Resampling to uniform voxel spacing (1.0 mm isotropic) Random cropping guided by positive/negative labels (RandCropByPosNegLabeld) Random flipping along x/y/z axes (probability = 0.10 each axis) Random 90-degree rotations (probability = 0.10) Random intensity shifts (± 10%, probability = 0.50)
Aortic calcium	Load 3D images and labels; ensure channels first Reorientation to RAS orientation Resampling to uniform voxel spacing (1.0 mm isotropic) Intensity normalization to [0,1] using window [-175, 250] (CT-specific) Foreground cropping based on intensity threshold Random cropping prioritizing rare classes (ratios [1,1,25] for classes 0,1,2 respectively) Random flipping along x/y/z axes (each axis probability = 0.10) Random 90-degree rotations (probability = 0.10) Random intensity shifts (offset ± 10%, probability = 0.50)
Liver, Spleen, Kidney, Pancreas	Random rotations Scaling Elastic deformations Mirroring Gamma correction Additive Gaussian noise

**Table 4:** A list of training hyperparameters and methods used for each of the segmentation models

Target Tissue/Organ	Optimizer	Loss Function	Regularization	Hyperparameters
Vertebral trabecular bone	Adam (learning rate = 0.0001, no weight decay)	PyTorch BCEWithLogitsLoss (binary cross-entropy loss with sigmoid activation)	Implicit via data augmentation (shift, scale, rotation); no explicit dropout or weight decay used	Epochs: 15 Number of output labels: 2 Weight initialization: Pretrained weights from TernausNet
Abdominal fat	Adam (learning rate = 0.00001, no weight decay)	PyTorch CrossEntropyLoss	Implicit via data augmentation (shift, scale, rotation with masking); no explicit dropout or weight decay used	Epochs: 25 Number of output labels: 3 Weight initialization: Pretrained weights from TernausNet; custom final layer initialized using PyTorch default initialization.
Muscle	AdamW (learning rate = 0.0001, weight decay = 1e-5), with cosine annealing learning rate scheduler (T_max = 300)	MONAI DiceLoss (includes background class, softmax activation, no one-hot encoding of labels)	Weight decay (L2 regularization, 1e-5) Dropout (drop_rate = 0.1, attention dropout = 0.1, stochastic depth/drop-path = 0.1) 3D data augmentation	Epochs: 100 Number of output labels: 2 Mixed-precision training via PyTorch AMP Weight initialization: Default PyTorch/MONAI
Aortic calcium	AdamW (learning rate = 0.0001, weight decay = 1e-5)	MONAI DiceCELoss (combination of Dice and CrossEntropy losses), with softmax activation and one-hot encoding of labels	Weight decay (L2 regularization, 1e-5) 3D data augmentation	Epochs: 30 Number of output labels: 2 Mixed-precision training via PyTorch AMP Weight initialization: Default PyTorch/MONAI
Liver, Spleen, Kidney, Pancreas	Stochastic Gradient Descent (SGD) with Nesterov momentum (momentum = 0.99), learning rate = 0.01 (with polynomial decay)	Combination of Dice loss and CrossEntropyLoss (multi-class segmentation)	Implicit via extensive data augmentation (rotation, scaling, elastic deformations, mirroring, gamma correction, Gaussian noise); no explicit dropout or weight decay specified	Epochs: Training until convergence Weight initialization: Default nnU-Net initialization strategy (adaptive initialization tailored to architecture specifics)

**Table 5:** Tissue and organ measurements performed by the OSCAR toolkit along with their units and accepted physiological ranges

Tissue/Organ	Biomarker	Units	Usable Range
Muscle (at L1/L3)	Cross-Sectional Area	cm^2^	25 to 500 cm^2^
	Density (attenuation) Mean/Median/Standard Deviation	CT Number (HU)	-50 HU to 200 HU
Aortic Calcium	Abdominal Agatston	unitless	0 to 40,000
	Total Aortic Agatston	unitless	0 to 40,000
Vertebral Trabecular Bone (at T10/T12/L1/L3)	Median Density (attenuation)	CT Number (HU)	-50 HU to 1200 HU
Adipose Tissue (at L1/L3)	Visceral Adipose Tissue Cross-Sectional Area	cm^2^	0 to 1200 cm^2^
	Subcutaneous Adipose Tissue Cross-Sectional Area	cm^2^	0.1 to 1000 cm^2^
	Total Adipose Tissue Cross-Sectional Area	cm^2^	0.1 to 1500 cm^2^
	Visceral to Subcutaneous Cross Sectional Area Ratio (VSR)	unitless	0.02 to 10.0
	Visceral Adipose Tissue Median Density	CT Number (HU)	-120 to -60 HU
	Subcutaneous Adipose Tissue Median Density	CT Number (HU)	-120 to -30 HU
Liver	Median Density (attenuation)	CT Number (HU)	-50 HU to 180 HU
	Volume	mL	100 to 5000 ml
Spleen	Median Density (attenuation)	CT Number (HU)	10 to 500 HU
	Volume	mL	50 to 6000 ml
Kidney	Median Density (attenuation)	CT Number (HU)	5 to 300 HU
	Volume	mL	50 to 750 ml
Pancreas	Median Density (attenuation)	CT Number (HU)	-10 to 260 HU
	Volume	mL	20 to 140 ml

### Body composition and biomarker measurements

Once the relevant organs and tissues are segmented, quantitative measurements can be derived with relative ease. Ideally, these measurements should approximate values that a trained observer, given sufficient time and appropriate tools, could obtain, thereby enhancing both explainability and interpretability. Commonly derived metrics include density (attenuation) values expressed in Hounsfield Units (HU), volumetric assessments, and cross-sectional areas measured at standard anatomical reference levels (e.g., at the L3 vertebra).

For density measurements, mean or median HU values are typically calculated from the segmented volume or a specific region of interest, with standard deviations reported where appropriate. Volumetric and cross-sectional area measurements are computed by combining the voxel dimensions of the input image with the count of voxels included in the segmentation. Notably, the use of cross-sectional area measurements mandates a consistent slice thickness to ensure reliable and comparable results.

A unique measure performed by the toolkit is the aortic Agatston score. This is measured by performing a slice-wise weighted sum of segmented calcium along the aorta as follows [37]:

$${\rm{Agatston}}\>{\rm{Score}} = {\sum \> _{Slices}}\left( {{\rm{Plaque}}\>{\rm{are}}{{\rm{a}}_{{\rm{Slice}}}} \times {\rm{Density}}\>{\rm{Facto}}{{\rm{r}}_{{\rm{Slice}}}}} \right)$$

where

$${\text{Density}}\:{\text{Facto}}{{\text{r}}_{{\text{Slice}}}} = \left\{ {\begin{array}{*{20}{c}}{\begin{array}{*{20}{c}}{1\:{\text{if}}\:{\text{peak}}\:{\text{HU}}\:{\text{is}}\:{\text{between}}\:130\:{\text{and}}\:199} \\ {2\:{\text{if}}\:{\text{peak}}\:{\text{HU}}\:{\text{is}}\:{\text{between}}\:200\:{\text{and}}\:299} \end{array}} \\ {\begin{array}{*{20}{c}}{3\:{\text{if}}\:{\text{peak}}\:{\text{HU}}\:{\text{is}}\:{\text{between}}\:300\:{\text{and}}\:399} \\ {\:4\:{\text{if}}\:{\text{peak}}\:{\text{HU}}\: \geqslant \:\:400} \end{array}} \end{array}} \right.$$

The pipeline measures two Agatston scores: 1. The abdominal Agatston, defined as the Agatston score measured from the diaphragmatic hiatus to the aortic bifurcation and 2) The total aortic Agatston, defined as the Agatston measured for the entire visible aorta.

For the various organs and tissues segmented by the OSCAR toolkit, these standardized measurement procedures yield robust, reproducible metrics suitable for subsequent analyses. To validate this reliability and clinical applicability of the automated pipeline, we evaluated potential failure modes and implemented error detection mechanisms in a prior study [1]. In that study, we identified several failure modes and corresponding error detection mechanisms by reviewing 11,699 examinations. In that cohort, at least one AI tool failed in 268 cases (2.3%), with segmentation errors being the most common failure mode. These errors were primarily due to overestimation of visceral fat area and underestimation of subcutaneous fat area. To detect such errors, we documented and established limits for each marker. These limits, presented in Table 5, were defined by reviewing histograms of data and examining outlier cases. The limits are set based on values above or below which no valid segmentations can be identified. The organ contrast enhancement is also used to automatically determine and validate presence or absence of intravenous contrast [38].

Scan parameters stored within DICOM headers can be valuable for implementing post-processing corrections or identifying potential failure modes. In addition to the discrete biomarkers captured in the results, key DICOM header fields are often recorded. These fields provide insights into the imaging process, including acquisition parameters, contrast characteristics, scanner specifications, patient attributes, and reconstruction details. The specific DICOM header fields recorded by the OSCAR toolkit are summarized in Table 6. The discrete output values.

**Table 6:** A list of DICOM header fields recorded by the OSCAR toolkit

	DICOM Field Name	DICOM Tag Number
Acquisition characteristics	X-Ray Tube Potential or kVp	(0018,0060)
	X-Ray Tube Current (mA)	(0018,1151)
	Exposure (mAs)	(0018,1152)
	Scan Options (helical or axial)	(0018,0022)
	Revolution Time (s)	(0018,9305)
	Spiral Pitch Factor	(0018,9311)
	Filter Type (body or head)	(0018,1160)
	Protocol Name	(0018,1030)
	Body Part Examined	(0018,0015)
	Laterality	(0020,0060)
Contrast characteristics	Contrast Bolus Agent	(0018,0010)
	Contrast Bolus Route	(0018,1040)
Scanner characteristics	System Manufacturer	(0008,0070)
	Manufacturer Model Name	(0008,1090)
	Software Versions	(0018,1020)
Patient characteristics	Patient Weight	(0010,1030)
Reconstruction characteristics	Image Type	(0008,0008)
	Convolution Kernel	(0018,1210)
	Reconstruction Diameter	(0018,1100)
	Slice thickness	(0018,0050)
	Spacing between slices	(0018,0088)

**Fig. 3:**
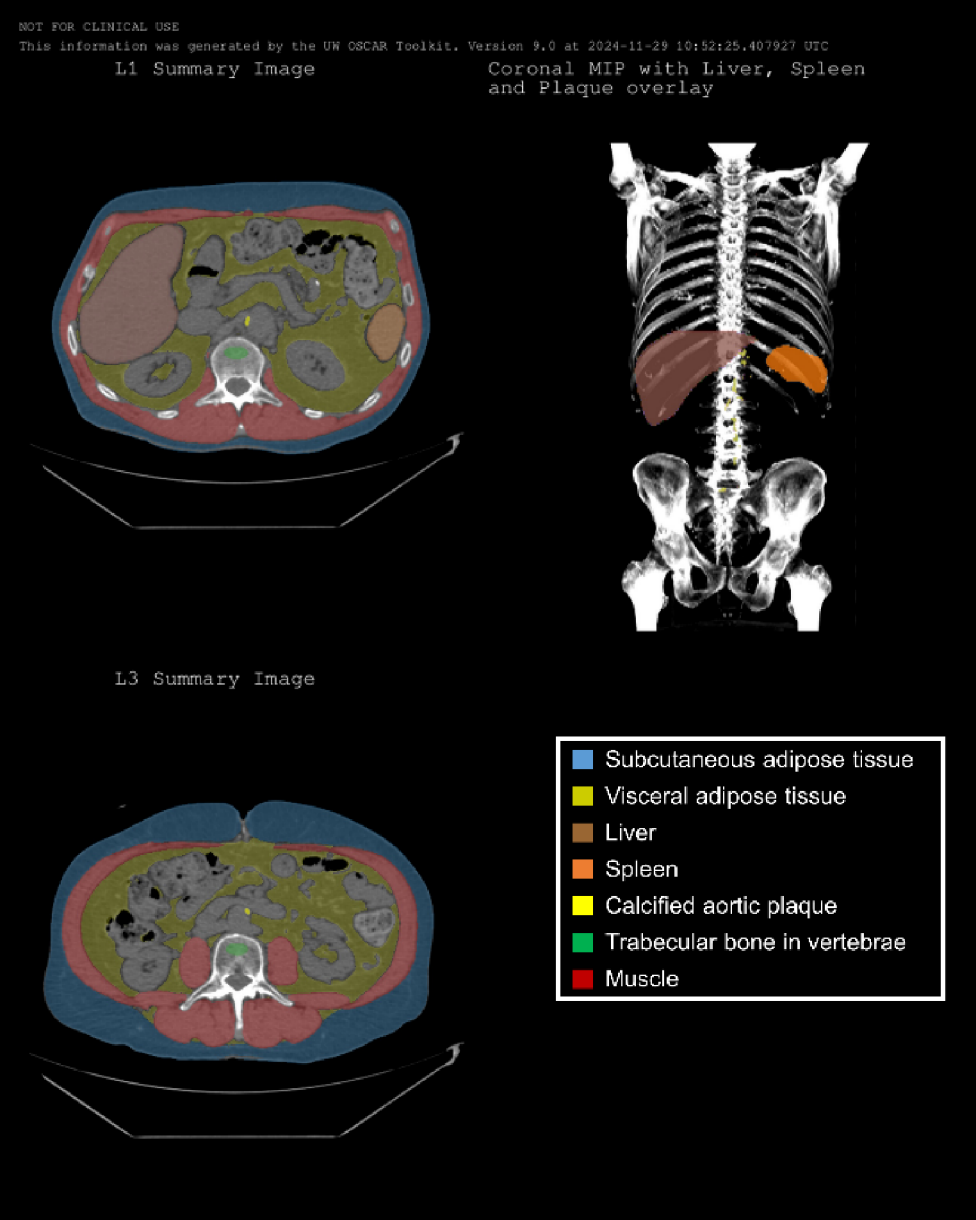
Sample quality assurance images from a 61-year-old male patient showing various organ and tissue segmentations

In addition to the discrete values output from the toolkit, visual outputs in the form of quality assurance images including segmentation overlays and coronal maximum intensity projections (MIP) images, are created to provide clear and interpretable results (see Fig. 3). These visualizations are created as DICOM objects with study and patient characteristics matching the original images so they can be shared back to the Picture Archiving and Communication Systems (PACS). These images can help clinicians quickly confirm the segmentations are correct and useable for measurement.

### Study selection, portability and compatibility

Study selection for the pipeline follows a structured, multi-step process. It begins with identifying procedures based on the manually entered “body part” field in our PACS database, targeting exams associated with the abdomen. Relevant examinations within a specified time frame are then retrieved using the DICOM *StudyDescription* field (0008,1030).

A series-level query is performed to apply inclusion criteria. A series is included if it: (1) contains at least 30 images; (2) represents a primary axial acquisition, excluding reformats or secondary captures based on the *ImageType* field (0008,0008); (3) has a slice thickness less than 7.5 mm, as defined by *SliceThickness* (0018,0050); and (4) does not involve dual-energy techniques such as virtual monochromatic or material basis imaging, as determined from the *SeriesDescription* field (0008,103E).

From the eligible series within each study, the pipeline selects a preferred series based on contrast phase priority, with preference in the following order: non-contrast, then portal venous, then delayed, and finally arterial. Within the selected phase, the series with the thinnest available slice thickness is chosen.

Ensuring portability, scalability, and computational efficiency involves leveraging containerization technologies, such as Docker [39], to encapsulate applications and their dependencies into portable containers. This method allows for consistent deployment across various environments, including cloud platforms, local servers, and edge devices [40]. Containerization ensures that the application behaves the same way regardless of the underlying infrastructure, which is crucial for maintaining the reliability and scalability of CT opportunistic screening tools which may be deployed in a variety of different ways and settings. By using containers, updates and patches can be applied seamlessly, and resources can be efficiently managed, facilitating the scaling of applications to meet varying demands. The OSCAR toolkit is packaged and distributed with collaborators as a Docker container which is maintained in a secure repository with version control. The current version of the tools is 9.3 (updated 02/28/2025); figures in this work were generated using version 9.0 (updated 11/25/2024).

A key consideration for both the practical adoption and the potential computational cost of implementing a toolkit like this in clinical practice is the amount of time required for processing. To evaluate processing time and assess the feasibility of clinical deployment, we applied the OSCAR toolkit to 1,242 renal donor CT exams. The experiment was conducted on a high-performance Linux server equipped with dual Intel Xeon Platinum 8468 CPUs (Intel Corp.), each with 48 cores and a base clock speed of 2.1 GHz, 1 TB of RAM, and four L40S GPUs (NVIDIA Inc.), though the pipeline was configured to run on a single GPU. Renal donor exams were selected as a representative use case because they are high-quality, standardized multiphase abdominal CT studies routinely acquired in healthy individuals, making them well-suited for evaluating end-to-end system performance under typical clinical imaging conditions. For this analysis, only the non-contrast phase (with 5 mm slices) was used. All exams were processed using this consistent hardware environment, enabling precise measurement of computational timing across key stages of the pipeline, including image segmentation, post-processing, and report generation. The timing results are shown in Table 7. The single most time consuming step is the organ segmentation with TotalSegmentator. Also notable is that the anatomical landmark identification is quick (~ 6 s) enabling a quick decision as to whether a given series is eligible or not.

**Table 7:** A summary of the time needed for each processing step in the OSCAR pipeline shown as a mean in seconds with the corresponding standard deviation as well as a percent of the total processing time

Processing Step	Mean (s)	Std. Dev. (s)	Mean % of total
Landmark Anatomical Landmark Identification	5.96	2.4	4
Organ Segmentation with TotalSegmentator	64.26	18.75	42.3
Aortic Plaque Segmentation	21.55	6.58	14.31
Muscle Segmentation	15.07	5.19	10.05
Trabecular Bone Segmentation	0.12	0.04	0.08
Fat Segmentation	0.15	0.05	0.1
Biomarker Measurement	7.23	3.2	4.83
DICOM Image and Report Generation	3.06	0.47	2.08
All Other Data Processing (resizing, copying, etc.)	33.03	6.6	22.26
Total Processing Time	150.43	29.79	100

## Discussion

This work advances the state-of-the-art in automated body composition analysis by integrating all key stages of the process into a comprehensive, reproducible pipeline. Unlike previous methodologies that often focused on isolated steps—such as segmentation or measurement—our solution unifies data normalization, anatomical landmark identification, segmentation, and biomarker extraction within a single framework. By reducing manual input and enhancing reproducibility, the pipeline is particularly well-suited for large-scale, multi-center studies where standardization and scalability are essential.

The segmentation tools implemented leverage machine learning and deep learning methods that have demonstrated strong robustness across diverse imaging conditions. Nonetheless, certain limitations persist. For instance, challenging patient anatomies or degraded image quality may reduce segmentation accuracy. Addressing these issues could involve domain adaptation strategies, model retraining with specialized subsets of data, and incorporating automated quality assurance checks to detect and mitigate problematic cases. In addition, ensuring quick and easy QA of the segmentations are available is crucial for clinical adoption or large-scale studies.

The extracted biomarkers, including volumetric, density, and cross-sectional area metrics for different tissues and solid organs have been validated in extensive work as useful and robust markers for opportunistic screening [1, 14, 33, 41–44]. Example use cases for these measurements include biological age characterization [7], risk stratification [45–49], longitudinal patient monitoring [50], and more [51–54]. By capturing scan and reconstruction parameters, our pipeline also enables refinement through downstream corrections and parameter adjustments, thus improving interpretability and ensuring that resulting metrics are both meaningful and methodologically transparent.

Finally, an emphasis on portability and compatibility allows this toolkit to function effectively across various scanner types, imaging protocols, and computational setups. This versatility not only lowers barriers to adoption but also sets the stage for widespread implementation, as exemplified by its integration within the OSCAR consortium. There are numerous potential applications for this toolkit in clinical workflows. Clinical adoption is likely to be facilitated by starting with workflows with clear clinical interventions or care pathways such as screening for osteoporosis [33] or referring high risk patients to preventative cardiology services [9]. An alternative starting point could be integrating the toolkit into existing clinical workflows, such as transplant patient prioritization, where it could provide objective frailty scoring to support decision-making [55].

The OSCAR toolkit used in this work is certainly not the only method by which body composition in abdominal CT is measured. There are several commercially available tools currently approved for various limited uses and several academic toolkits developed globally. Specifically, the Comp2Comp toolkit [56], the BOA toolkit [57], the ABC toolkit [58], and CompositIA [59]. Each toolkit has a slightly different list of segmentation tools and many of them leverage tools like TotalSegmentator [19] for segmentation of various organs. Notably, these tools also diverge in label definitions and metrics reported (e.g., whether intramuscular fat is treated separately or included within muscle) and few currently benchmark performance by prognostic value and instead focus on quantitative evaluation using segmentation metrics like Dice scores. These discrepancies complicate direct comparison, underscoring the need for harmonized definitions and evaluation against clinical outcomes. A format such as a multi-center validation study or a grand challenge to validate and benchmark body composition toolkits in terms of prognostic value would be an important step in term of understanding the true strengths and weaknesses of each toolkit.

This work has several limitations. First, the pace of AI-based image segmentation tool development is rapidly evolving. In practice, this means there is an ever-growing set of tools available to perform segmentation and analysis, making it extremely challenging to determine an optimal model and approach to a task like this. In addition, the models in this toolkit require GPU hardware to run efficiently, which may lead to considerable implementation costs and limit accessibility in lower-resource settings. Another key limitation is the reliance on retrospective imaging data from a single health system (albeit including diverse outreach sites), which may impact generalizability to other populations, scanners, or protocols. Furthermore, while segmentation quality was assessed through failure mode analysis and manual review, comprehensive evaluation of clinical validity and downstream utility remains an important next step.

Future work will focus on refining the pipeline for faster performance, expanding to other anatomical regions, and integrating with health informatics platforms (such as electronic health records and clinical decision support systems) thereby enhancing the pipeline’s clinical and research utility. Additional directions include prospective validation in diverse patient populations and care settings, implementation of real-time refinement of segmentations by human observers or complementary AI tools, and further exploration of active learning approaches to continuously improve model performance in deployment.

## Conclusion

We have presented a fully automated AI-driven pipeline for body composition analysis from abdominal CT scans, demonstrating its capacity to serve as a robust and versatile foundation for multi-center studies. By standardizing inputs, automating segmentation, and extracting clinically relevant biomarkers, the methodology fosters reproducibility, interpretability, and scalability. Its adaptability to diverse imaging conditions and incorporation within the OSCAR consortium underscores its potential to advance large-scale research endeavors and clinical decision-making in the field of body composition analysis.

## Data Availability

No datasets were generated or analysed during the current study.
